# Supplementary material for: Observed different impacts of potential tree restoration on local surface and air temperature
Source: Nat Commun. 2025 Mar 8;16:2335. doi: 10.1038/s41467-025-57606-y (PMC11890605; doi:10.1038/s41467-025-57606-y)
Supplement: Supplementary file 1 — Supplementary Information [file 41467_2025_57606_MOESM1_ESM.pdf]

Supplementary information for:

Observed different impacts of potential tree restoration on local surface and air temperature

Authors:

Yitao Li[1,2], Zhao-Liang Li[3*], Hua Wu[4], Xiangyang Liu[3], Xu Lian[5], Menglin Si[3], Jing Li[3], Chenghu Zhou[6], Ronglin Tang[1,2], Sibo Duan[3], Wei Zhao[7], Pei Leng[3], Xiaoning Song[2], Qian Shi[8], Enyu Zhao[9], Caixia Gao[10]

Affiliations:

[1]State Key Laboratory of Resources and Environment Information System, Institute of Geographic Sciences and Natural Resources Research, Chinese Academy of Sciences; Beijing 100101, China.

[2]University of Chinese Academy of Sciences; Beijing 100049, China.

[3]State Key Laboratory of Efficient Utilization of Arable Land in Northern China/Institute of Agricultural Resources and Regional Planning, Chinese Academy of Agricultural Sciences; Beijing 100081, China.

[4]School of Resources and Environment, University of Electronic Science and Technology of China; Chengdu, 611731, China.

[5]Department of Earth and Environmental Engineering, Columbia University; New York, 10027, United States of America.

[6]Center for Ocean Remote Sensing of Southern Marine Science and Engineering Guangdong Laboratory (Guangzhou), Guangzhou Institute of Geography, Guangdong Academy of Sciences; Guangzhou 510070, China.

[7]Institute of Mountain Hazards and Environment, Chinese Academy of Sciences; Chengdu 610041, China.

[8]School of Geography and Planning, Sun Yat-sen University; Guangzhou 510275, China.

[9]Information Science and Technology College, Dalian Maritime University; Dalian 116026, China.

[10]Key Laboratory of Quantitative Remote Sensing Information Technology, Aerospace Information Research Institute, Chinese Academy of Sciences; Beijing 100094, China.

[*]Correspondence: lizhaoliang@caas.cn;

supplementary items list:

Supplementary Figures 1–13
Supplementary Table 1
Supplementary Reference

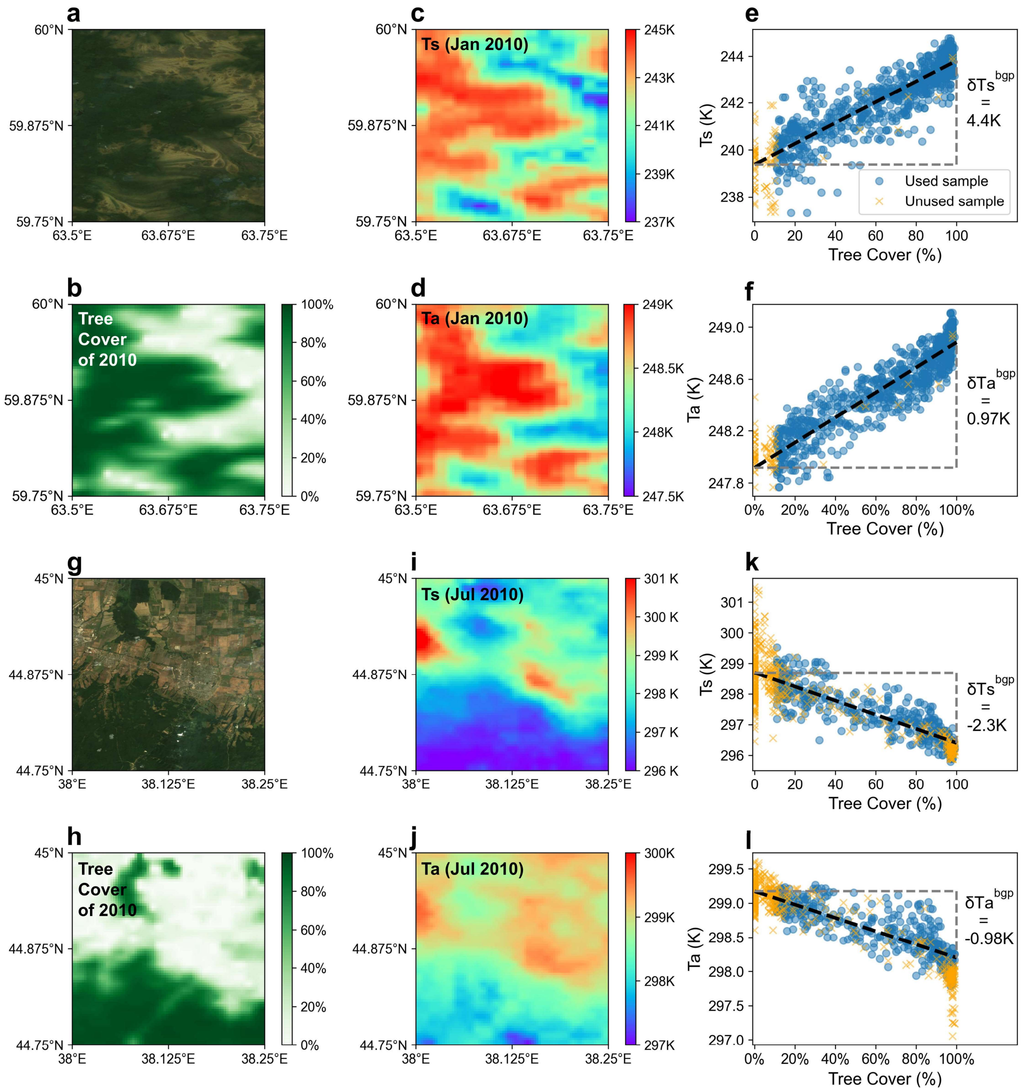

Supplementary Figure 1. Schematic representation of the methodology for estimating land surface temperature (Ts) or air temperature (Ta) sensitivity (δTs^{bph} and δTa^{bph}). (a–f) The example grid (59.75°–60°N, 63.5°–63.75°E) with positive sensitivities in January. (a) True color image of the 0.25° grid. (b) GLOBMAP 2010 tree cover map. (c) Mean Ts of January 2010. (d) Mean T_a of January 2010. (e) Linear regression for estimating δTs^{bph} of the grid. (f) Linear regression for estimating δTa^{bph} of the grid. (g–l) Similar to (a–f), but for the other grid (44.75°–45°N, 38°–38.25°E) with negative sensitives in July.

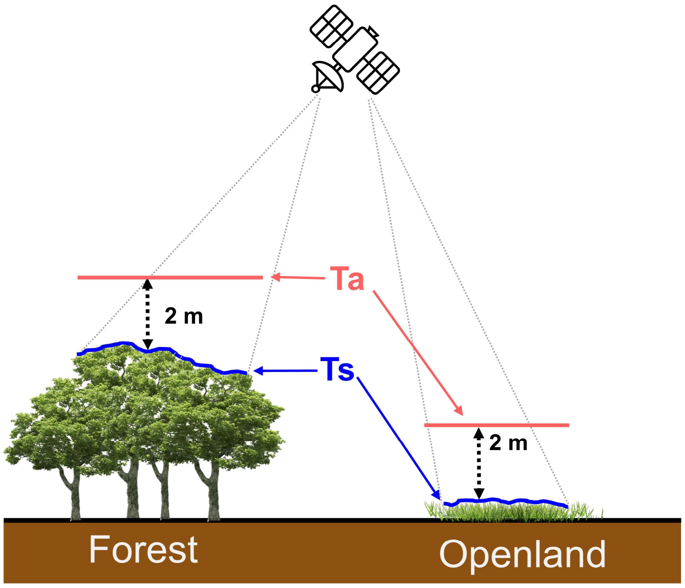

Supplementary Figure 2. Schematic illustration of evaluated land surface temperature (Ts) and near surface air temperature (Ta). The evaluated Ta indicates the air temperature of about 2 m above the land surface. Here, land surface indicates the interface layer between different land surface components and the atmosphere (e.g., vegetation canopy). Thus, for forested areas, Ta refers to the air temperature of about 2 m above the tree canopy. While for openlands, the reference plane is approximately the ground.

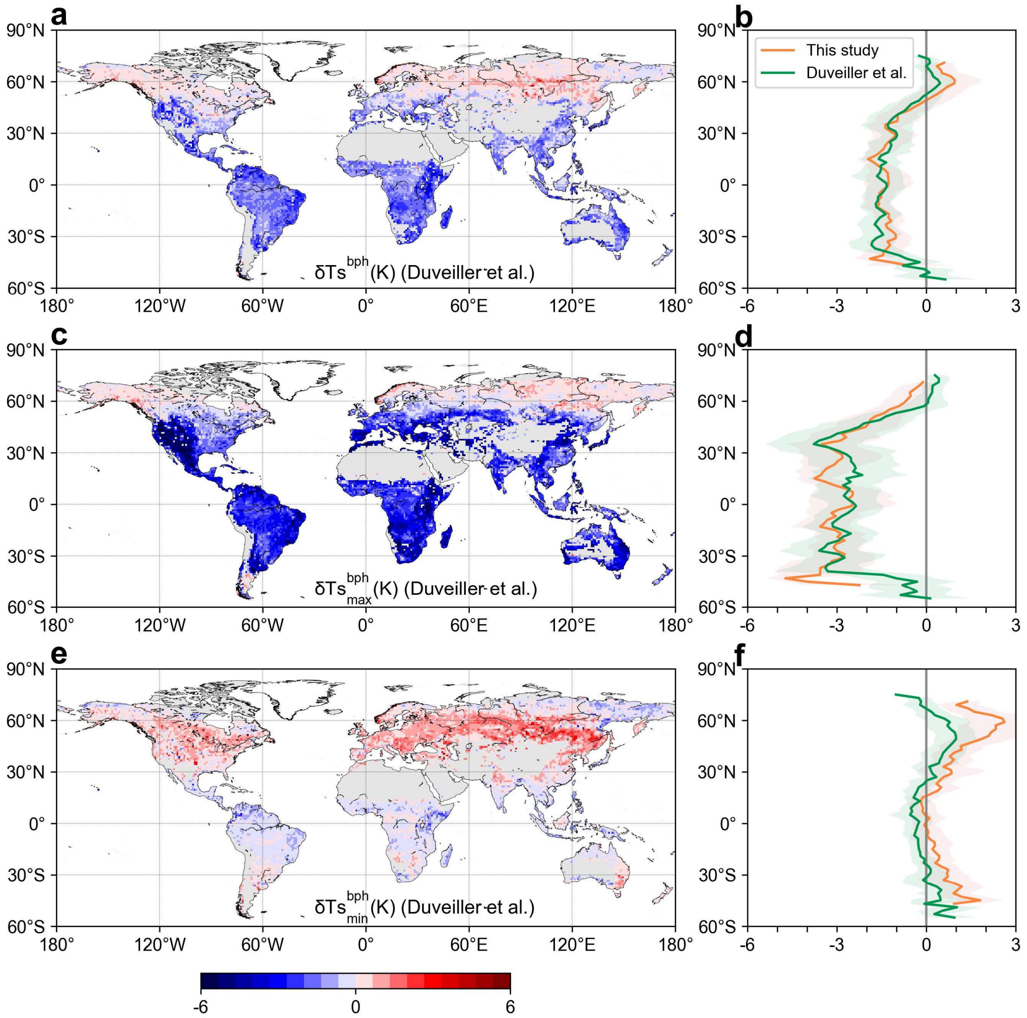

Supplementary Figure 3. Comparison of annual land surface temperature sensitivity to forestation ($\delta \mathrm{Ts}^{\mathrm{bph}}$) between our results and the previous study[1]. (a) Global pattern of $\delta \mathrm{Ts}^{\mathrm{bph}}$ from the previous study. (b) Comparison of latitudinal pattern of $\delta \mathrm{Ts}^{\mathrm{bph}}$ from this study and previous study. (c) and (d) Same with (a) and (b), but for maximum temperature sensitivity ($\delta \mathrm{Ts}^{\mathrm{bph}}_{\mathrm{max}}$). (e) and (f) Same with (a) and (b), but for minimum temperature sensitivity ($\delta \mathrm{Ts}^{\mathrm{bph}}_{\mathrm{min}}$).

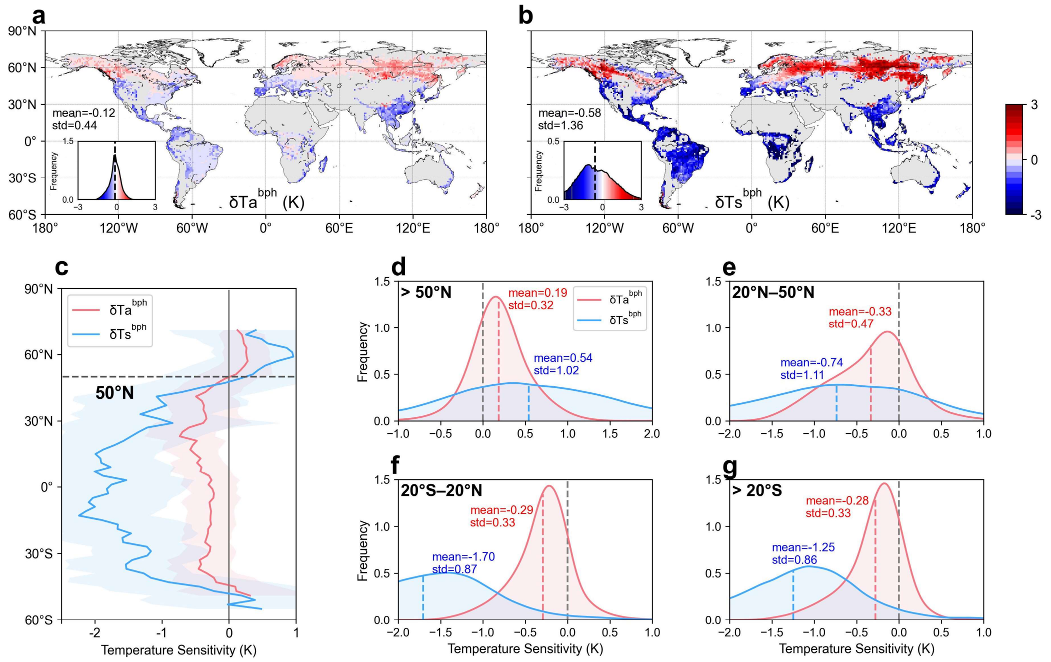

Supplementary Figure 4. Annual mean temperature sensitivity to the full tree cover restoration derived from Global Forest Change (GFC) tree cover data. (a) Global pattern of air temperature sensitivity ($\delta \mathrm{Ta}^{\mathrm{bph}}$). (b) Global pattern of land surface temperature sensitivity ($\delta \mathrm{Ts}^{\mathrm{bph}}$). (c) The variation of $\delta \mathrm{Ta}^{\mathrm{bph}}$ and $\delta \mathrm{Ts}^{\mathrm{bph}}$ across latitudinal bands, with the shaded area indicating the standard deviation across space. (d–g) The probability density of $\delta \mathrm{Ta}^{\mathrm{bph}}$ and $\delta \mathrm{Ts}^{\mathrm{bph}}$ across northern high-latitudes (>50°N), northern mid-latitudes (20°–50°N), tropics (20°S–20°N) and southern mid-latitudes (>20°S).

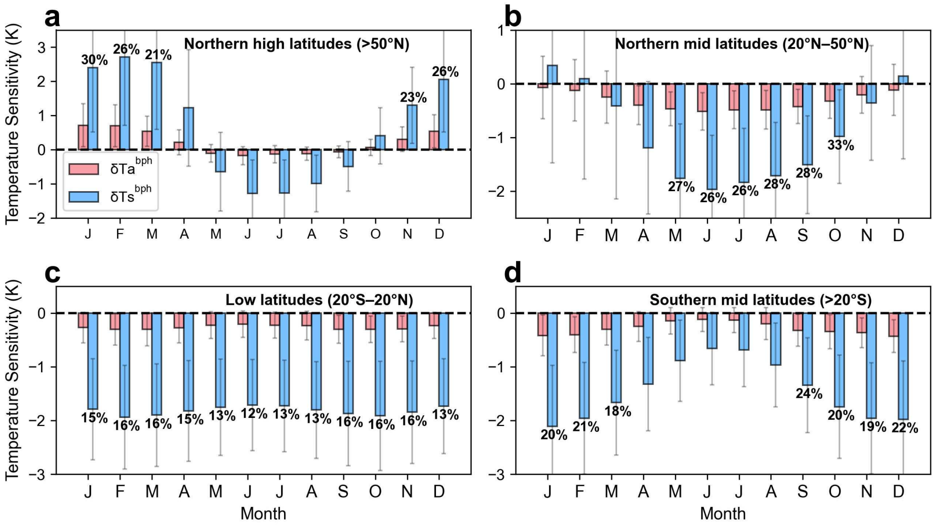

Supplementary Figure 5. Seasonally pattern of air temperature sensitivity (δTa^{bph}) and land surface temperature sensitivity (δTs^{bph}) in (a) northern high-latitudes, (b) northern mid-latitudes, (c) low-latitudes, and (d) southern mid-latitudes. The error bars indicate the spatial standard deviation. The figures below or above the bars indicate the ratio of δTa^{bph} to δTs^{bph}. Only months with significant temperature effects are shown.

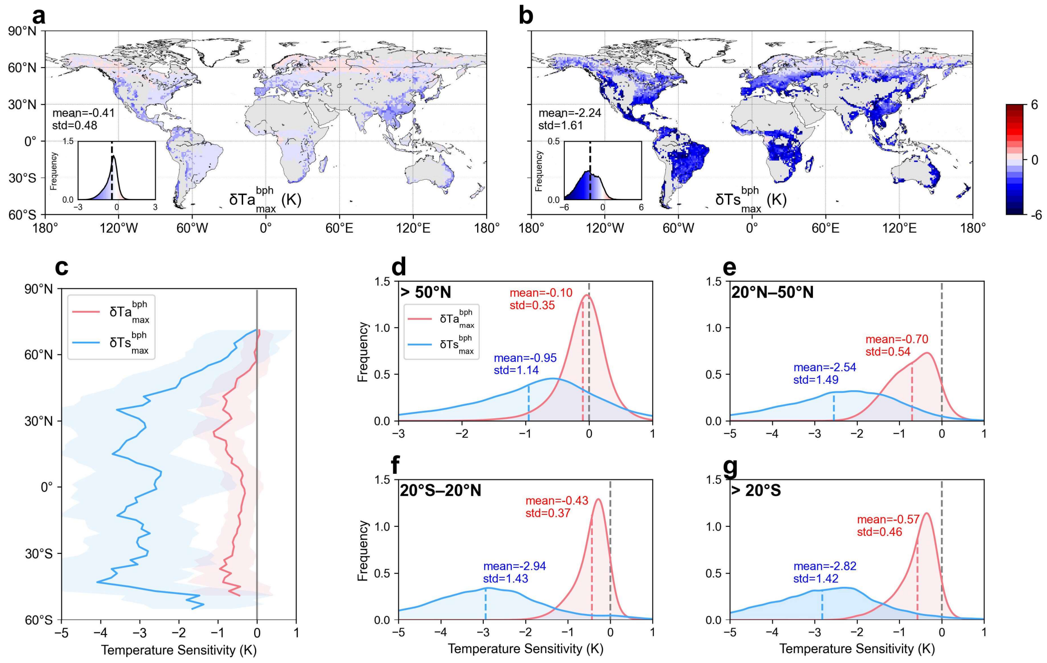

Supplementary Figure 6. Annual mean daily maximum air temperature and land surface temperature sensitivity to the full tree cover restoration. (a) Global pattern of daily maximum air temperature sensitivity ($\delta \mathrm{Ta}_{\max}^{\mathrm{bph}}$). (b) Global pattern of daily max land surface temperature sensitivity ($\delta \mathrm{Ts}_{\max}^{\mathrm{bph}}$). (c) The variation of $\delta \mathrm{Ta}_{\max}^{\mathrm{bph}}$ and $\delta \mathrm{Ts}_{\max}^{\mathrm{bph}}$ across latitudinal bins, shaded area indicates the standard deviation. (d–g) The probability density of $\delta \mathrm{Ta}_{\max}^{\mathrm{bph}}$ and $\delta \mathrm{Ts}_{\max}^{\mathrm{bph}}$ across northern-high latitudes (>50°N), northern mid-latitudes (20°–50°N), tropics (20°S–20°N) and southern mid-latitudes (>20°S).

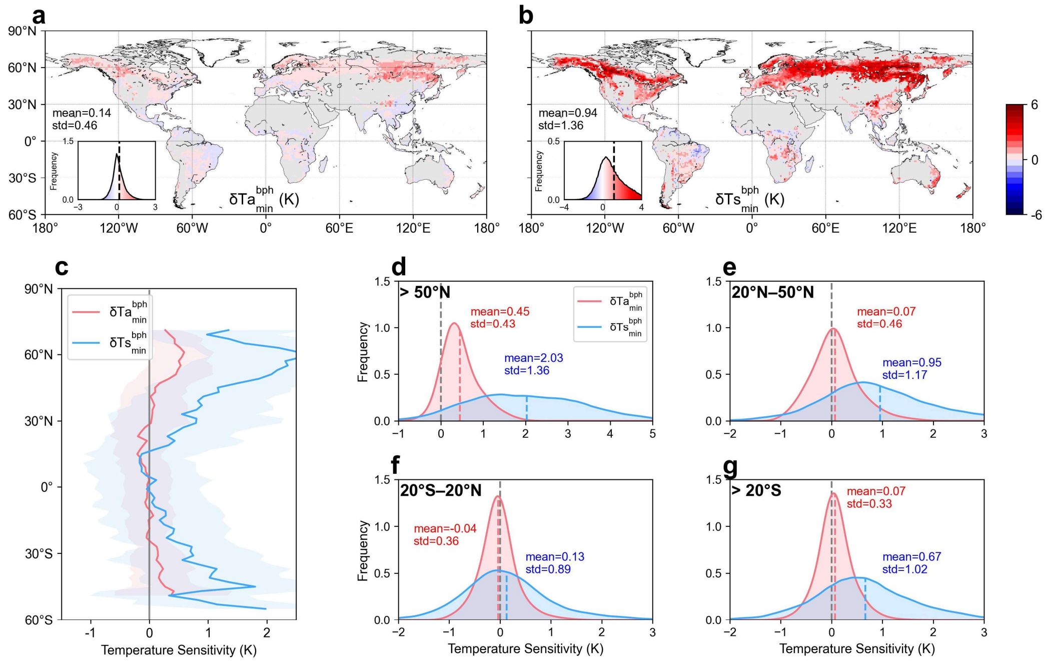

Supplementary Figure 7. Annual mean daily minimum air temperature and land surface temperature sensitivity to the full tree cover restoration. (a) Global pattern of daily minimum air temperature sensitivity (δTa^{bph}_{min}). (b) Global pattern of daily minimum land surface temperature sensitivity (δTs^{bph}_{min}). (c) The variation of δTa^{bph}_{min} and δTs^{bph}_{min} across latitudinal bins, shaded area indicates the standard deviation. (d–g) The probability density ofδTa^{bph}_{min} and δTs^{bph}_{min} across northern-high latitudes (>50°N), northern mid-latitudes (20°–50°N), tropics (20°S–20°N) and southern mid-latitudes (>20°S).

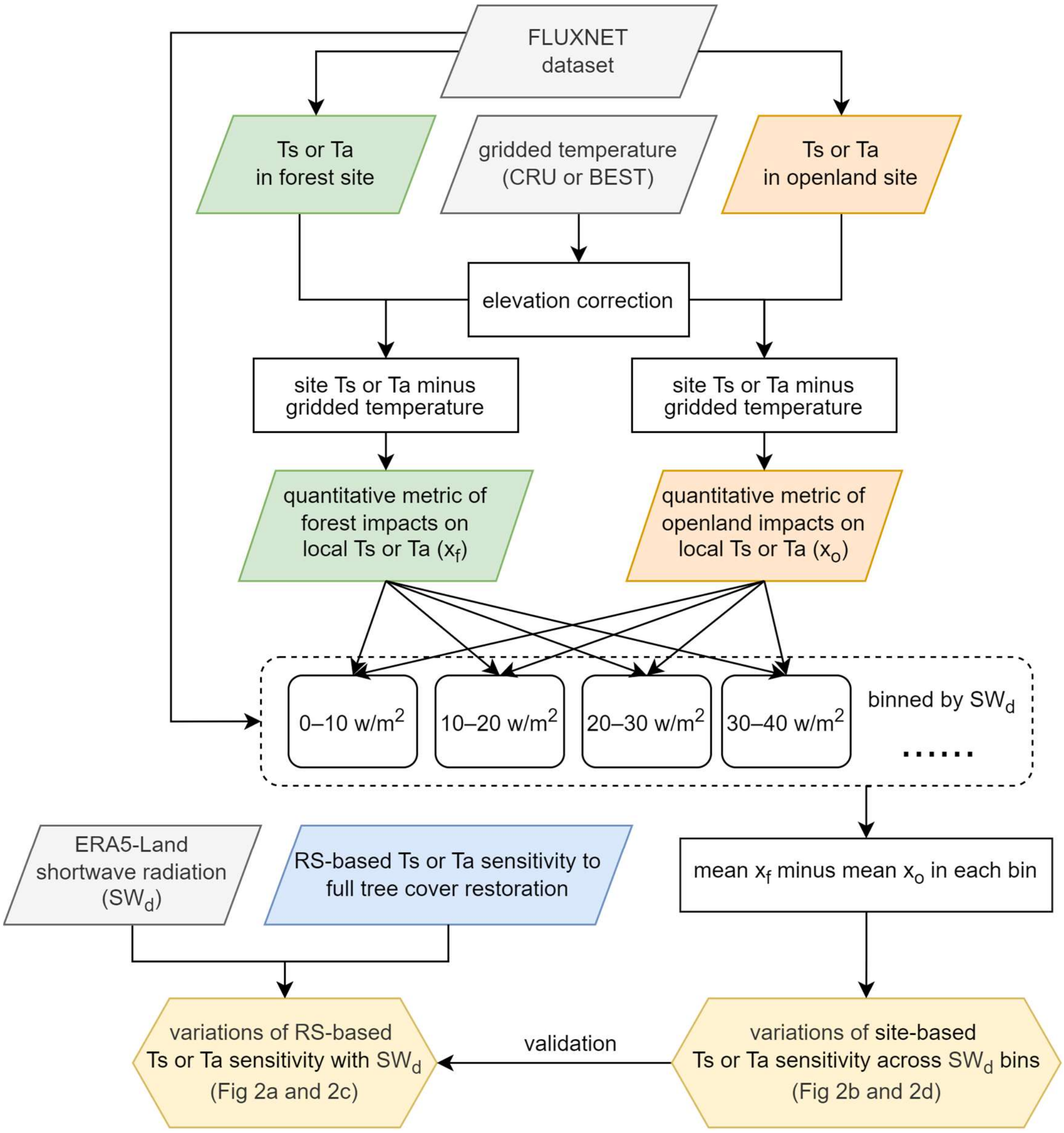

Supplementary Figure 8. Flow chart of the remote sensing (RS)-based local temperature effect validation using FLUXNET and gridded temperature data. Abbreviations: Ta, air temperature; Ts, land surface temperature; CRU, Climatic Research Unit; BEST, Berkeley Earth Surface Temperatures.

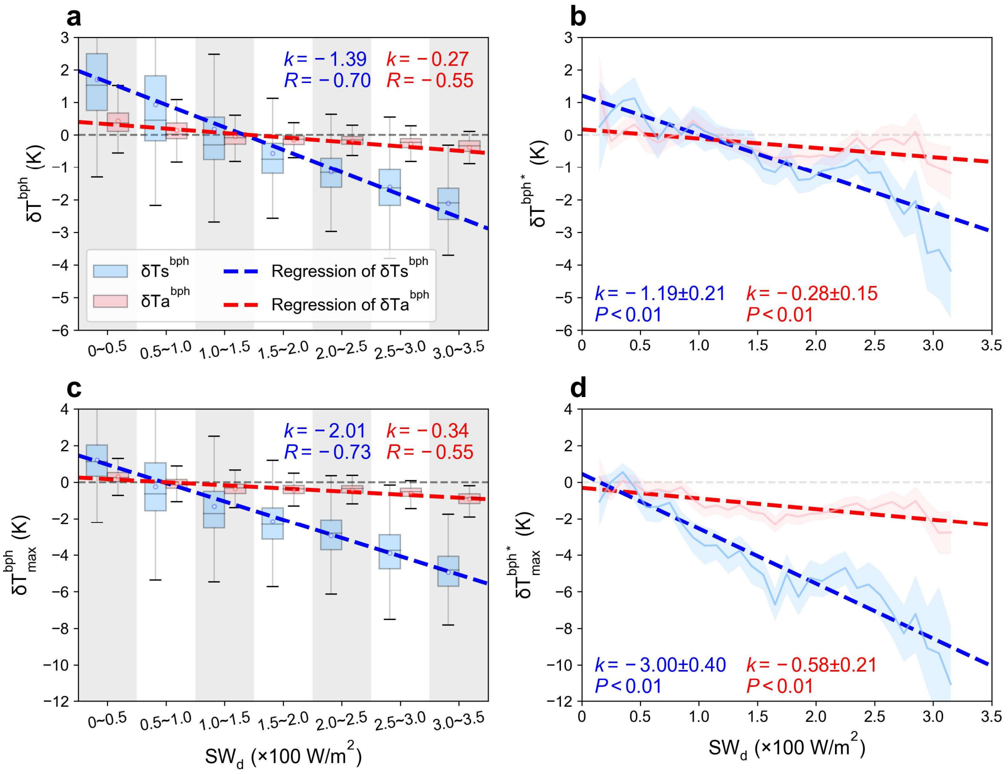

Supplementary Figure 9. Validation of the monthly land surface temperature and air temperature sensitivities using Berkeley Earth Surface Temperatures (BEST) data as the background. (a) Remote sensing-based relationships between mean temperature sensitivities (δTs^{bph} and δTa^{bph}) and background shortwave radiation (SW_d). (b) FLUXNET-based relationships between the mean temperature sensitivities (δTs^{bph*} and δTa^{bph*}) and SW_d. The shaded area indicates the standard error for the mean sensitivity within each SW_d bin. (c) Same as (a), but for the maximum temperature sensitivities derived from remote sensing data (δTs^{bph}_{max} and δTa^{bph}_{max}). (d) Same as (b), but for the maximum temperature sensitivities from FLUXNET measurements (δTs^{bph*}_{max} and δTa^{bph*}_{max}).

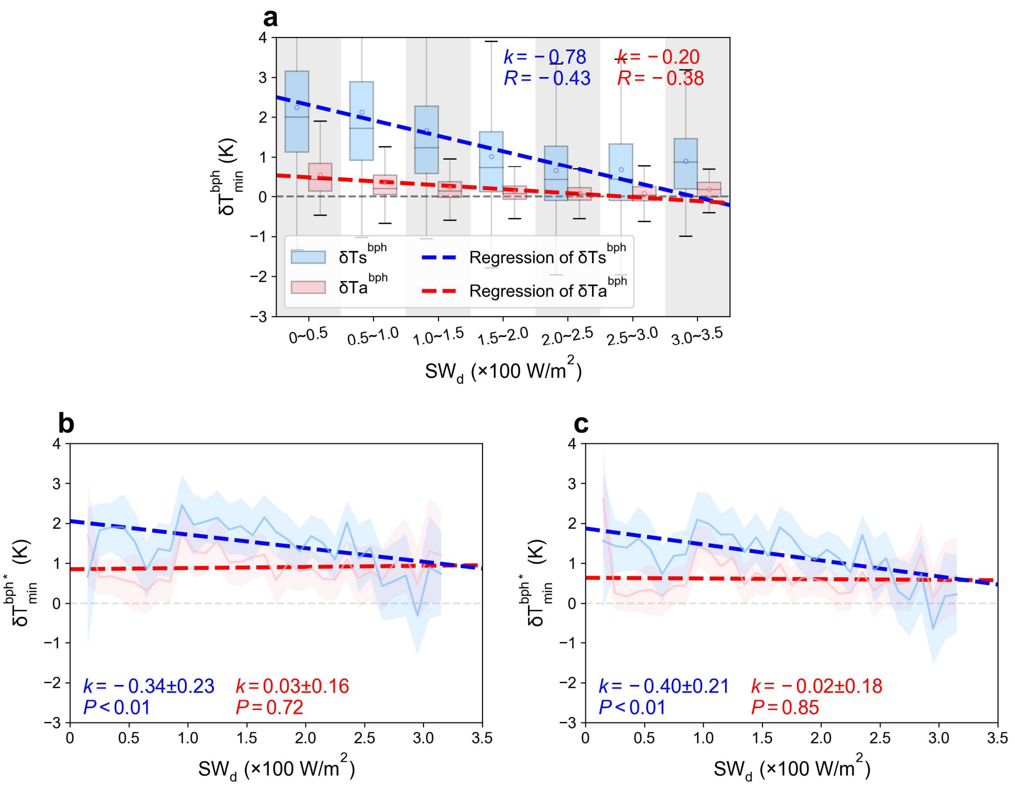

Supplementary Figure 10. Validation of the monthly minimum land surface temperature and air temperature sensitivities. (a) Remote sensing-based relationships between minimum temperature sensitivities (δTs^{bph}_{min} and δTa^{bph}_{min}) and background shortwave radiation (SW_d). (b) FLUXNET-based relationships between minimum temperature sensitivities (δTs^{bph*}_{min} and δTa^{bph*}_{min}) with SW_d, using Climatic Research Unit (CRU) temperature data to exclude the impact of macro-climate background. (c) Same as (b), but the Berkeley Earth Surface Temperatures (BEST) data are used to exclude the impact of macro-climate background.

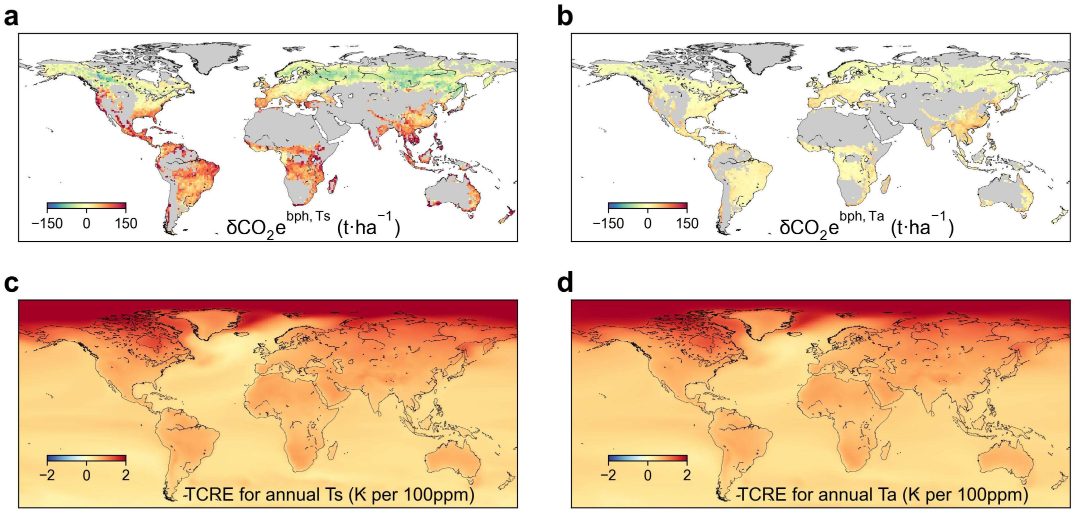

Supplementary Figure 11. Equivalent CO_2 uptake induced by biophysical Ts and Ta effects of potential tree restoration ($\delta CO_2 e^{bph,Ts}$ and $\delta CO_2 e^{bph,Ta}$). (a) Global map of $\delta CO_2 e^{bph,Ts}$. (b) Global map of $\delta CO_2 e^{bph,Ta}$. (c) Transient climate response to cumulative emissions (TCRE) of CO_2 for annual Ts. (d) Transient climate response to cumulative emissions (TCRE) of CO_2 for annual Ta.

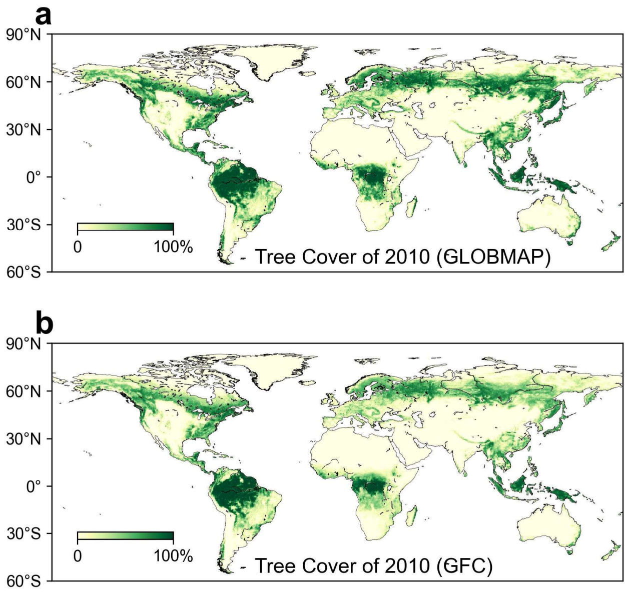

Supplementary Figure 12. Global maps of fractional tree cover from (a) GLOBMAP and (b) Global Forest Change (GFC) datasets.

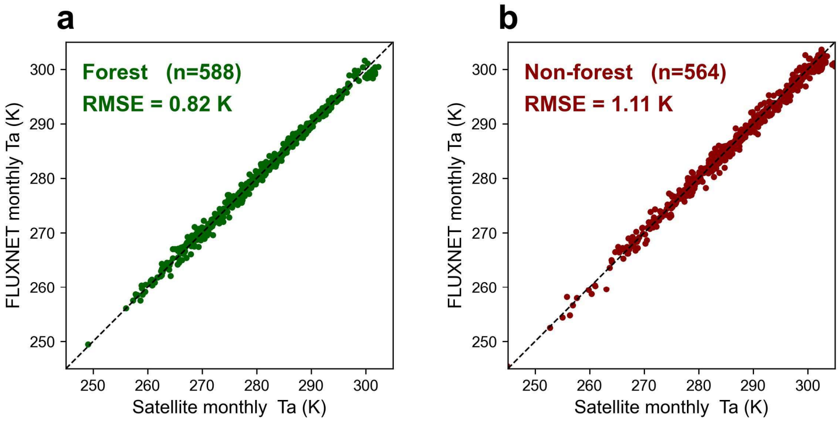

Supplementary Figure 13. Validation of satellite monthly mean air temperature (Ta) of 2010. (a) Scatter plot between satellite monthly Ta and FLUXNET monthly Ta measurements in forest sites. (b) Similar to (a), but for non-forest sites. Abbreviation: RMSE, root mean squared error.

Supplementary Table 1. Detailed information of the FLUXNET sites used in the sensitivity validation. The first column gives the site name; the second and third columns give the location information of each site; the fourth column gives the land cover type (ENF: evergreen needleleaf forests; EBF: evergreen broadleaf forests; DBF: deciduous broadleaf forests; MF: mixed forests; CRO: croplands; GRA: grasslands; OSH: open shrublands; CSH: closed shrublands; SAV: savannas; WSA: woody savannas; WET: permanent wetlands); the fifth column gives the whether the site was used in the attributional analysis.

Name	Latitude	Longitude	Landcover	Used in Attribution Analysis
AT-Neu	47.11667	11.3175	GRA	√
AU-ASM	-22.283	133.249	ENF	√
AU-Cpr	-34.0021	140.5891	SAV	√
AU-Cum	-33.6152	150.7236	EBF	√
AU-DaP	-14.0633	131.3181	GRA	
AU-DaS	-14.1593	131.3881	SAV	√
AU-Dry	-15.2588	132.3706	SAV	√
AU-Emr	-23.8587	148.4746	GRA	√
AU-Gin	-31.3764	115.7138	WSA	
AU-GWW	-30.1913	120.6541	SAV	√
AU-How	-12.4943	131.1523	WSA	
AU-Lox	-34.4704	140.6551	DBF	√
AU-RDF	-14.5636	132.4776	WSA	
AU-Rig	-36.6499	145.5759	GRA	√
AU-Rob	-17.1175	145.6301	EBF	
AU-Stp	-17.1507	133.3502	GRA	√
AU-TTE	-22.287	133.64	OSH	√
AU-Wac	-37.4259	145.1878	EBF	
AU-Whr	-36.6732	145.0294	EBF	√
AU-Wom	-37.4222	144.0944	EBF	√
AU-Ync	-34.9893	146.2907	GRA	√
BE-Bra	51.30761	4.51984	MF	√
BE-Lon	50.5516	4.74613	CRO	
BR-Sa3	-3.01803	-54.9714	EBF	
CA-Gro	48.2167	-82.1556	MF	
CA-Oas	53.62889	-106.198	DBF	
CA-Obs	53.98717	-105.118	ENF	
CA-Qfo	49.6925	-74.3421	ENF	√
CA-SF1	54.48503	-105.818	ENF	√
CA-SF2	54.25392	-105.878	ENF	
CA-SF3	54.09156	-106.005	OSH	√
CA-TP4	42.71016	-80.3574	ENF	√
CA-TPD	42.63533	-80.5577	DBF	√

CH-Cha	47.21022	8.41044	GRA	√
CH-Dav	46.81533	9.85591	ENF	√
CH-Fru	47.11583	8.53778	GRA	√
CH-Oe1	47.28583	7.73194	GRA	
CN-Cng	44.5934	123.5092	GRA	
CZ-BK1	49.50213	18.53686	ENF	
CZ-BK2	49.49443	18.54285	GRA	
CZ-wet	49.02465	14.77035	WET	
DE-Geb	51.1001	10.9143	CRO	
DE-Gri	50.95004	13.51259	GRA	√
DE-Hai	51.07917	10.453	DBF	√
DE-Kli	50.89306	13.52238	CRO	
DE-Lkb	49.09962	13.30467	ENF	
DE-Lnf	51.32822	10.3678	DBF	√
DE-Obe	50.78666	13.72129	ENF	√
DE-RuR	50.62191	6.30413	GRA	√
DE-RuS	50.86591	6.44717	CRO	
DE-SfN	47.80639	11.3275	WET	
DE-Tha	50.96235	13.56516	ENF	√
DK-Sor	55.48587	11.64464	DBF	√
FI-Hyy	61.84741	24.29477	ENF	
FI-Lom	67.99724	24.20918	WET	
FR-Gri	48.84422	1.95191	CRO	
FR-LBr	44.71711	-0.7693	ENF	
FR-Pue	43.7413	3.5957	EBF	
GF-Guy	5.27877	-52.9249	EBF	
GH-Ank	5.26854	-2.69421	EBF	
IT-BCi	40.52375	14.95744	CRO	
IT-CA1	42.38041	12.02656	DBF	
IT-CA2	42.37722	12.02604	CRO	
IT-CA3	42.38	12.0222	DBF	
IT-Col	41.84936	13.58814	DBF	
IT-Isp	45.81264	8.63358	DBF	
IT-La2	45.9542	11.2853	ENF	
IT-Lav	45.9562	11.28132	ENF	√
IT-MBo	46.01468	11.04583	GRA	√
IT-Noe	40.60618	8.15117	CSH	
IT-Ren	46.58686	11.43369	ENF	√
IT-Ro1	42.40812	11.93001	DBF	
IT-Ro2	42.39026	11.92093	DBF	
IT-SR2	43.73203	10.29095	ENF	
IT-SRo	43.72786	10.28444	ENF	
IT-Tor	45.84444	7.57806	GRA	√
JP-SMF	35.2617	137.0788	MF	
MY-PSO	2.973	102.3062	EBF	
NL-Hor	52.24035	5.0713	GRA	√

NL-Loo	52.16658	5.74356	ENF	√
NO-Adv	78.186	15.923	WET	
RU-Che	68.61304	161.3414	WET	
RU-Cok	70.82914	147.4943	OSH	
RU-Fyo	56.46153	32.92208	ENF	
SE-St1	68.35415	19.05033	WET	
US-AR1	36.4267	-99.42	GRA	√
US-AR2	36.6358	-99.5975	GRA	√
US-ARM	36.6058	-97.4888	CRO	
US-CRT	41.6285	-83.3471	CRO	
US-GLE	41.36653	-106.24	ENF	√
US-Goo	34.2547	-89.8735	GRA	√
US-Ivo	68.4865	-155.75	WET	
US-Los	46.0827	-89.9792	WET	
US-Me2	44.4523	-121.557	ENF	
US-Me3	44.3154	-121.608	ENF	
US-Me6	44.32328	-121.608	ENF	
US-MMS	39.3232	-86.4131	DBF	√
US-Ne1	41.16506	-96.4766	CRO	
US-Ne2	41.16487	-96.4701	CRO	
US-Ne3	41.17967	-96.4397	CRO	
US-NR1	40.0329	-105.546	ENF	√
US-Oho	41.5545	-83.8438	DBF	√
US-Prr	65.12367	-147.488	ENF	
US-SRC	31.9083	-110.84	OSH	
US-SRG	31.78938	-110.828	GRA	
US-SRM	31.8214	-110.866	WSA	
US-Syv	46.242	-89.3477	MF	
US-Tw4	38.10298	-121.641	WET	
US-UMB	45.5598	-84.7138	DBF	
US-Var	38.4133	-120.951	GRA	
US-WCr	45.8059	-90.0799	DBF	√
US-Whs	31.7438	-110.052	OSH	
US-Wkg	31.7365	-109.942	GRA	
US-WPT	41.46464	-82.9962	WET	
ZA-Kru	-25.0197	31.4969	SAV	√
ZM-Mon	-15.4378	23.25278	DBF	√

Supplementary Reference

1. Duveiller, G., Hooker, J. & Cescatti, A. A dataset mapping the potential biophysical effects of vegetation cover change. *Sci. Data* **5**, 1–15 (2018).
